# Genetic susceptibility to *Candida* infections

**DOI:** 10.1002/emmm.201201678

**Published:** 2013-04-30

**Authors:** Sanne P Smeekens, Frank L van de Veerdonk, Bart Jan Kullberg, Mihai G Netea

**Affiliations:** Department of Medicine, Radboud University Nijmegen Medical Centre and Nijmegen Institute for Infection, Inflammation, and Immunity (N4i)Nijmegen, The Netherlands

**Keywords:** *Candida albicans*, primary immunodeficiencies, interferon gamma, interleukin-17, disease susceptibility

## Abstract

*Candida* spp. are medically important fungi causing severe mucosal and life-threatening invasive infections, especially in immunocompromised hosts. However, not all individuals at risk develop *Candida* infections, and it is believed that genetic variation plays an important role in host susceptibility. On the one hand, severe fungal infections are associated with monogenic primary immunodeficiencies such as defects in *STAT1*, *STAT3* or *CARD9*, recently discovered as novel clinical entities. On the other hand, more common polymorphisms in genes of the immune system have also been associated with fungal infections such as recurrent vulvovaginal candidiasis and candidemia. The discovery of the genetic susceptibility to *Candida* infections can lead to a better understanding of the pathogenesis of the disease, as well as to the design of novel immunotherapeutic strategies. This review is part of the review series on host-pathogen interactions. See more reviews from this series.

## Infections with *Candida* species

*Candida* spp, especially *Candida albicans*, are commensal fungi that reside on the skin, mucosa and gastrointestinal tract of 30 to 50% of healthy individuals at any given time, with everyone being colonized at a certain moment of his/her lifetime (Brown & Netea, 2007). Although *C. albicans* is not pathogenic under normal host conditions, it can cause severe mucosal or systemic infections when host defense is compromised.

### Mucosal infections

Mucosal infections affect the skin and mucous membranes. Common sites for these superficial infections are the mouth, vagina, external ear, skin and nails, of which oral candidiasis is the most common (Odds, 1988). Mucosal infections are usually sporadic, but some patients experience severe and recurrent infections of the skin and oropharyngeal cavities termed chronic mucocutaneous candidiasis (CMC). In addition, most women suffer at least once in their lifetime from vulvovaginal candidiasis, while up to 8% of them have recurrent infections (Sobel, 2007).

### Systemic infections

In contrast to mucosal candidiasis which is highly prevalent but does not cause high mortality, systemic infections are life threatening, with mortality rates reaching up to 26–60% (Das et al, 2011). When the organisms enter the blood stream they can invade deep tissues and organs such as brain, heart and kidneys. Considering the number of patients diagnosed each year, *Candida* has emerged in the recent decades as one of the most important pathogens in sepsis, causing significant morbidity and mortality. Moreover, mortality due to these severe infections has not been significantly changed in the last decade, despite the introduction of potent antifungals such as azoles and echinocandins (Fortún et al, 2012). It is currently believed that only a combination of standard antimycotic treatment with adjuvant immunotherapy may significantly improve the outcome of fungal infections, and both immunological and genetic studies are needed to accomplish the necessary understanding of the pathogenesis of these infections.

## *Candida albicans* host defense

**Figure 1:**
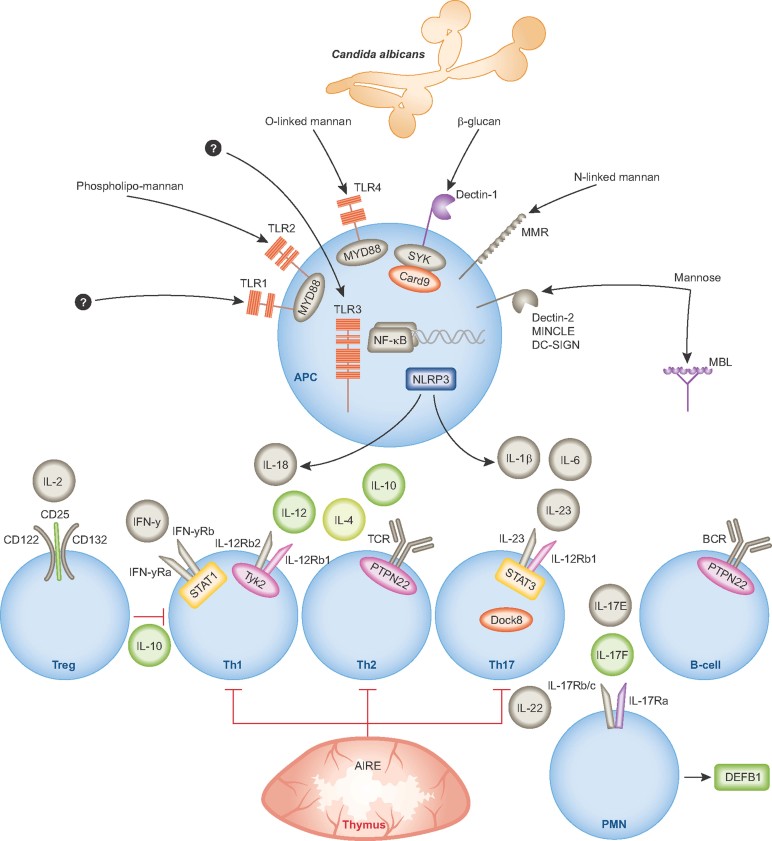
Schematic overview of the anti-*Candida albicans* immune response When *Candida* is recognized by Toll-like receptors (TLRs) and C-type lectin receptors, the production of cytokines is initiated through activation of transcription factors like NF-κB. IL-1β and IL-18 first need to be cleaved by the NLRP3 inflammasome before they can be secreted. IL-2 is involved in the differentiation of all effector T-cells. The IL-2 receptor is highly expressed on regulatory T-cells (T_reg_). IL-12 and IL-18 promote the differentiation of T helper 1 (Th1) cells, with IFN-γ being their main product. IL-4 and IL-10 promote the differentiation of Th2 cells, while IL-10 can also suppress Th1 cells. IL-1β, IL-6 and IL-23 drive the development of Th17 cells. DOCK8 is involved in the maintenance of Th17 cells. IL-17 promotes the recruitment of neutrophils, which have tissue protective effects by the production of beta-defensins. Cytokines are recognized by cytokine receptors, which use several adaptor molecules like STAT1, STAT3 and TYK2. PTPN22 is involved in B- and T-cell receptor signaling. Components with mutations and/or genetic variation known to be associated with *Candida* infection are shown in color. APC: antigen presenting cell, BCR: B-cell receptor, CARD9: caspase recruitment domain 9, DC-SIGN: dendritic cell-specific ICAM-grapping non-integrin, MBL: mannose binding lectin, MMR: macrophage mannose receptor, NLRP3: NACHT, LRR and PYD domains-containing protein 3, TCR: T-cell receptor, TLR: Toll-like receptor.

The *C. albicans* cell wall can be divided into two distinct layers: the inner layer consisting mainly of polysaccharides like chitin, 1,3-β-glucans and 1,6-β-glucans, and the outer layer consisting mainly of proteins that are heavily mannosylated with mannan side-chains. These pathogen-associated molecular patterns (PAMPs) can be recognized by several pathogen recognition receptors (PRRs), such as the Toll-like receptors (TLRs) and C-type lectins (CLRs) on the surface of antigen presenting cells (APCs). TLR2 recognizes phospholipomannans (Jouault et al, 2003), and TLR4 recognizes *O*-linked mannans (Netea et al, 2006). *N*-linked mannans are recognized by the macrophage mannose receptor (MMR) (Netea et al, 2006), with other CLRs which can recognize mannose residues being Dectin-2 (McGreal et al, 2006), Mincle (Wells et al, 2008), DC-specific ICAM-grapping non-integrin (DC-SIGN) (Cambi et al, 2003) and the soluble receptor mannose-binding lectin (MBL) (Brouwer et al, 2008). The CLR Dectin-1 recognizes β-glucan (Brown & Gordon, 2001) (Fig 1)

When a PRR recognizes its corresponding ligand, adaptor molecules engage with the receptor. Different types of PRRs use different adaptor molecules, which transduce a signal by activating a kinase cascade, in order to induce the transcription of proinflammatory cytokines. Dectin-1 signals through Syk (Rogers et al, 2005) and caspase recruitment domain 9 (CARD9) (Gross et al, 2006). Dectin-1 can induce cytokine production independently of other receptors, as well as synergize with TLRs for an optimal stimulation of the cell. When ligands are recognized by TLRs, signals are transduced intracellularily through adaptor proteins like myeloid differentiation factor (MYD)88. Subsequently, a mitogen-activated protein kinase (MAPK) response is activated leading to the nuclear translocation of transcription factors like NF-κB and c-Jun, inducing the transcription of cytokines and chemokines (Akira et al, 2006). Interestingly, depending on the fungal burden and amount of hyphae formation a second MAPK phase, consisting of MKP1 and c-Fos activation, can be initiated, further promoting proinflammatory responses (Moyes et al, 2010).

The recognition of *C. albicans* by cells of the innate immune system will lead to phagocytosis (Heinsbroek et al, 2008) and killing of the invading pathogen. At the same time, the production of cytokines is induced that on the one hand activate inflammation, and on the other hand engage and direct the adaptive immune response. Activation of the caspase-1 component of the inflammasome, mediated by the intracellular activation of the NOD-like receptor NLRP3, is a central event leading to the processing of pro-IL-1β and pro-IL-18 into their respective bioactive cytokines, directing the induction of Th17 and Th1 responses, respectively (Cheng et al, 2011; Lalor et al, 2011). IFN-γ production by Th1 cells, and IL-17 production by Th17 cells are important characteristics of the *Candida*-induced immune response (Netea et al, 2008). Inflammasome and Th17 activation is considered to be a central event for the discrimination of colonization *versus* invasion with *C. albicans* at the level of the mucosa (Gow et al, 2011).

## General risk factors for *Candida* infections

*C. albicans* is an opportunistic fungal pathogen. In healthy individuals, the immune response will usually clear infections, but an immunocompromised immune system causes a significant increase in the risk for *Candida* infections. Das et al demonstrated that 92% of *Candida* bloodstream infections are preceded by a course of broad-spectrum antibiotics (Das et al, 2011), which suppress the growth of the normal bacterial flora and eliminates natural antagonism of fungal colonization of the mucosa. There are several other examples in which *Candida* acts as an opportunistic pathogen. For example, almost all AIDS and oncologic patients with neutropenia suffer from oropharyngeal candidiasis (Grabar et al, 2008; Viscoli et al, 1999). Furthermore, 41% of patients undergoing hematopoietic stem cell transplantation, for which the immune system is destroyed beforehand, suffer from one or more bloodstream infections within the first ten years after transplantation, 4% of which are caused by *Candida* spp. The crude mortality rate associated with these *Candida*-infections is 42% (Ortega et al, 2005). Also patients with systemic lupus erythematosus (SLE), which are treated with glucocorticoids and other immunosuppressive agents, have an increased risk for invasive fungal infections (IFI), which are predominantly caused by *Candida* spp. (Fan et al, 2012).

**Glossary**

**Autosomal-dominant**

Mode of inheritance in which the presence of only one copy of a gene on one of the 22 autosomal–non-sex chromosomes, will result in the phenotypic expression of that gene.

**Candidemia**

The presence of *Candida* species in the blood.

**Candidiasis**

Fungal infection with any of the *Candida* species. Includes candidemia (in case of systemic infection).

**Chronic mucocutaneous candidiasis (CMC)**

An immune disorder characterized by chronic infections with *Candida* that are limited to mucosal surfaces, skin and nails.

**Genetic variation**

Variations of genomes between members of species or between groups of species. Includes SNP (in case it is a common genetic variant), mutation (in case it is a rare genetic variant) and copy-number variation.

**Immunocompromised**

State in which the immune system is not functioning properly, increasing susceptibility to infection.

**Immunodeficiency**

A state in which the immune system's ability to fight infectious disease is compromised or entirely absent.

**Immune paralysis**

A state in which induction of tolerance is due to injection of large amounts of antigen that remains poorly metabolized.

**Neutropenia**

An immune disorder characterized by an abnormally low level of neutrophils.

**Pathogenesis**

The mechanism by which the disease is caused.

**Pathogen recognition receptors (PRRs)**

Proteins expressed by cells of the innate immune system, which recognize pathogen-associated molecular patterns (PAMPs) from microbial pathogens.

**Polymorphism**

Having multiple alleles of a gene within a population, usually linked to different phenotypes.

**Single nucleotide polymorphism (SNP)**

DNA sequence variation occurring when a single nucleotide in the genome differs between members of a biological species or paired chromosomes in an individual.

Not only a weakened immune system increases the risk for *Candida* infections, also the extent to which individuals are colonized with pathogens plays a significant role in the development of candidiasis. Candidiasis typically affects patients with prolonged hospitalization. Fifty-one percent of *Candida* blood-stream infections is associated with being admitted to the ICU (Das et al, 2011). The mean time of onset of systemic *Candida* infections is 22 days after hospitalization (Wisplinghoff et al, 2004). Furthermore, when barriers to the outside world are damaged or breached by medical devices or surgery, this creates a portal of entry for pathogens like *C. albicans*. For instance, major abdominal surgery poses an increased risk for systemic *Candida* infections, which is underlined by the observation that in a cohort of 107 patients with candidemia, 50% underwent recent surgery (Das et al, 2011). Another factor contributing to systemic candidiasis is the fact that *Candida* spp. can form biofilms on many medical devices like central venous catheters (CVC), contact lenses, intrauterine devices (IUDs) (Donlan & Costerton, 2002) and pacemakers (Glöckner, 2011). *Candida* can even cause prosthetic joint infections, although they are considered to be rare (Springer & Chatterjee, 2012). Indeed, neonates on the intensive care unit (ICU) with a central line often suffer from infections, with the third most causative pathogen being *Candida* spp. Fortunately this incidence is decreasing due to the use of anti-fungal prophylaxis (Chitnis et al, 2012).

## Genetic risk factors for *Candida* infections

In spite of the important role played by these risk factors, they do not explain all *Candida* infections, and only a minority of individuals at risk will eventually develop a fungal infection. It is therefore believed that also genetic factors must play an important role in determining the susceptibility to *Candida* infections. Indeed, mutations in single genes were found to be responsible for severe *Candida* infections in several primary immunodeficiencies that display the clinical picture of monogenetic disorders. However, these disorders are rare, and in the majority of patients no sole causative genetic factor can be found. In most patients a combination of gene polymorphisms and/or environmental factors will determine whether a patient will develop a *Candida* infection. The genetic susceptibility to more common *Candida* infections such as RVVC or candidemia is likely polygenic, but the understanding of the genetic factors that determine it is nevertheless crucial for future immunotherapeutic approaches in these patients.

### Monogenetic disorders

Several monogenetic disorders have been described in the literature to be associated with an increased susceptibility to fungal infections. Glocker et al described that a homozygous mutation in the *CARD9* gene, coding for a protein downstream of Dectin-1, results in an increased susceptibility to both mucosal and invasive *Candida* infections (Glocker et al, 2009; Lanternier et al, 2012). Disease severity in these patients is likely explained by the fact that CARD9 is also involved in the downstream signaling of several other CLR receptors, such as Dectin-2 and Mincle (Robinson et al, 2009; Saijo et al, 2010; Strasser et al, 2012; Yamasaki et al, 2008), implying that CARD9 is a central mediator of anti-*Candida* host defense.

Another monogenetic disorder that results in an important primary immunodeficiency associated with *Candida* infections is CMC. Both autosomal recessive and autosomal dominant variants of the disease have been described. Mutations in the CC-domain of *STAT1*, a signaling molecule downstream of the type I and type II IFN receptor (Darnell et al, 1994), but also IL-23 and IL-12 receptors (as heterodimer with STAT3 or STAT4), have recently been demonstrated to be the main cause of autosomal-dominant CMC (van de Veerdonk et al, 2011), and these findings were confirmed by several other research groups (Depner et al, 2012; Hirata et al, 2012; Liu et al, 2011; Martinez-Martinez et al, 2012; Moreira et al, 2012; Okada et al, 2012; Smeekens et al, 2011). In addition to *STAT1* mutations, Puel et al demonstrated the presence of mutations in *IL-17RA* and *IL-17F* in some unexplained CMC cases (Puel et al, 2012). In contrast, patients with autosomal recessive autoimmune polyendocrinopathy candidiasis ectodermal dystrophy (APECED) not only suffer from CMC, but also experience autoimmune phenomena (Lilic, 2002). APECED has been linked to mutations in the *autoimmune regulator* (*AIRE*) gene (Björses et al, 1998) that result in a loss-of-function phenotype, causing the production of neutralizing autoantibodies against important cytokines with antifungal properties such as IL-17E, IL-17F and IL-22 (Puel et al, 2010).

Another monogenetic defect resulting in a primary immunodeficiency syndrome associated with *Candida* infections of the skin is hyper-IgE syndrome (HIES). HIES was first described as Job's syndrome and is characterized by high serum IgE levels, eczema, recurrent mucosal infections with *C. albicans*, and skin and pulmonary infections with *Staphylococcus aureus* (Davis et al, 1966). There are a number of mutations known to be associated with HIES. Several mutations have been found in *STAT3* (Holland et al, 2007; Minegishi et al, 2007), a signaling molecule downstream of the IL-23 receptor, resulting in absent IL-17 production (de Beaucoudrey et al, 2008; Ma et al, 2008; Milner et al, 2008; Sharfe et al, 1997). Other genes which have been associated with HIES include *dedicator of cytokinesis* (*DOCK*)*8 that codes* for a protein involved in Th17 polarization (Engelhardt et al, 2009) and *TYK2* (Minegishi et al, 2006), coding for a Janus kinase (JAK) downstream of the IL-12 receptor (Shimoda et al, 2000). All in all, defective Th17 responses underlie both CMC and HIES, two immunodeficiencies associated with severe, chronic, mucosal *Candida* infections. This emphasizes the importance of the Th17 response in mucosal *Candida* immunity.

Also mutations in genes coding for cytokines and their receptors have been described to be associated with *Candida* infections. For example, IL-12Rb1 deficiency has been linked to mucocutaneous *Candida* infections, and these patients also have increased susceptibility for invasive candidiasis (Rodríguez-Gallego et al, 2012). Sharfe et al described a patient with a deletion in the *CD25* gene, suffering from esophageal candidiasis. CD25 is the α-subunit of the IL-2 receptor, which is constitutively expressed on T regulatory cells (Sakaguchi et al, 1995). Furthermore, IL-2 is involved in the differentiation of effector T cells. Although Sharfe et al only described a single patient, this again emphasizes the importance of T cells in the anti-*Candida* host response. A complete overview of monogenetic disorders causing fungal infections is depicted in Table 1 and Fig 1.

**Table 1:** Monogenetic disorders

Gene	Mutation	Mode of inheritance	Phenotype	Disease	Refs.
AIRE	R257X	Autosomal-dominant	Autoantibodies against IL-17 and IL-22	CMC	Nagamine et al (1997), Pearce et al (1998)
CARD9	Q295X	Autosomal-recessive	Reduced TNF-α production and Th17 cells	CMC	Glocker et al (2009)
	Q289X R101C	Autosomal-recessive	Reduced Th17 responses	Invasive dermamtophytic disease	Lanternier et al (2012)
CD25	Deletion (60–64)	Autosomal recessive	Reduced number of CD4+ cells	*Candida* esophagitis	Sharfe et al (1997)
DOCK8	Multiple deletions and point mutations	Autosomal-recessive	Reduced Th17 cells	Hyper IgE syndrome	Engelhardt et al (2009)
IL-12Rb1	Multiple point mutations	Autosomal-recessive	Low levels of IFN-γ	Mucosal candidiasis	Rodríguez-Gallego et al (2012)
IL-17RA	Q284X	Autosomal-recessive	Absent IL-6 and GRO-a production	CMC	Puel et al (2011)
IL-17F	S65L	Autosomal-dominant	Reduced IL-6 and GRO-a production	CMC	Puel et al (2011)
STAT1	R274W A267V	Autosomal-dominant	Reduced IL-17, IL-22 and IFN-γ production	CMC	van de Veerdonk et al (2011)
STAT3	Multiple point mutations	Autosomal-dominant	Reduced IL-17 production	HIES	Holland et al (2007)
TYK2	Deletion (550–553)	Autosomal-recessive	Reduced Th1 and Type I IFN responses	HIES	Minegishi et al (2006)

### Common genetic variants and susceptibility to *Candida* infections

Despite the presence of primary immunodeficiency syndromes with fungal infections, the vast majority of fungal infections is not present in these individuals, but are common diseases with a polygenic pattern of increased susceptibility. Several studies have been published showing a link between genetic variation and an increased risk for *Candida* infections, with different genetic pattern being discerned between mucosal and systemic candidiasis. An example of this dichotomy is the role of a *Dectin-1* polymorphism for susceptibility to mucosal, but not systemic, candidiasis. We have recently described a family in which its members suffered from recurrent vulvo-vaginal candidiasis (RVVC) and onychomycosis. Their symptoms could be explained by an early stop codon in *Dectin-1* (Y238X) that resulted in defective β-glucan recognition and Th17 responses. Interestingly, this polymorphism is present in up to 8% of the Europeans and up to 40% of some sub-Saharan African populations (Ferwerda et al, 2009), being associated with mucosal *Candida* colonization and treatment in haematopoetic patients (Plantinga et al, 2009), but not with systemic candidiasis (Rosentul et al, 2011).

Genetic variation localized in other PRRs, such as the TLRs, has also been associated with an increased susceptibility to fungal infections. Three single nucleotide polymorphisms (SNPs) in the *TLR1* gene have been shown to influence susceptibility to candidemia, presumably mediated by decreased levels of IL-8 and IFN-γ (Plantinga et al, 2012). However, these findings need to be replicated in independent studies, and it is unclear which component of *Candida* is recognized by TLR1. A similar observation has been made for TLR2 and TLR4, which recognize phospholipomannans and *O*-linked mannans, respectively. The R753Q *TLR2* polymorphism increased the risk for candidemia in one small study through decreased IFN-γ and IL-8 levels (Woehrle et al, 2008), and two SNPs in the TLR4 gene were shown to be a risk factor for candidemia through increased IL-10 production (Van der Graaf et al, 2006), but these observations were not replicated in a larger study of patients (Plantinga et al, 2012). Nahum et al suggested that the L412F *TLR3* polymorphism increases the risk for CMC, an effect mediated by decreased IFN-γ production (Nahum et al, 2011). Furthermore, variable number of tandem repeats in *MBL2* gene that codes for the soluble PRR MBL has been linked to RVVC in two separate studies (Babula et al, 2003; Giraldo et al, 2007). Finally, length polymorphisms in the *NLPR3* gene, coding for the receptor subunit of the NLRP3 inflammasome, can increase the risk for RVVC (Lev-Sagie et al, 2009).

In addition to the first step of pathogen recognition, genetic variation in several cytokines has been linked to an increased risk for *Candida* infections. Choi et al demonstrated that the −1089T/G, −589C/T and the −33C/T polymorphisms in *IL-4* are associated with chronic disseminated candidiasis (Choi et al, 2003). Interestingly, the −589T/C SNP has also been demonstrated to pose a risk for RVVC (Babula et al, 2005). The −1082A/G polymorphism in the anti-inflammatory cytokine gene *IL-10* and the 274INS/DEL polymorphism in *IL-12b*, are associated with persisting candidemia (Johnson et al, 2012). These data strongly suggest that the balance between pro- and anti-inflammatory cytokines represent an important component of host defense against both mucosal and systemic candidiasis.

The −44C/G polymorphism in *DEFB1*, coding for beta-defensin 1, is correlated with increased *Candida* carriage (Jurevic et al, 2003). The exact underlying mechanism is unclear, but in general beta-defensins are secreted by neutrophils and epithelial cells and contribute to epithelial immunity. The R620W polymorphism in PTPN22, a protein involved in T-cell and B-cell receptor signaling, was suggested to be associated with an increased risk for CMC. Although the potential mechanism of this association is unclear (Nahum et al, 2008). A complete overview of common genetic variants associated with fungal infection is depicted in Table 2 and Fig 1.

**Table 2:** Common genetic variants

Gene	SNP (rs-number)	Phenotype	Disease	Refs.
Dectin-1	Y238X **(rs16910526)**	Decreased IL-1β and Th17 responses	*Candida* colonization	Plantinga et al (2009)
DEFB1	−44C/G (rs1800972)	Unknown	*Candida* carriage	Jurevic et al (2003)
IL-4	−589T/C (rs2243250)	Increased vaginal IL-4, reduced NO and MBL levels	RVVC	Babula et al (2005)
	−1098T/G (rs2243248), −589C/T (rs2243250), −33C/T (rs2070874)	Unknown	Chronic disseminated candidiasis	Choi et al (2003)
IL-10	−1082A/G (rs1800896)	Higher *Candida*-induced IL-10 production	Persisting candidemia	Johnson et al (2012)
IL-12B	2724INS/DEL (rs17860508)	Lower *Candida*-induced IFN-γ production	Persisting candidemia	Johnson et al (2012)
MBL2	Variable number of tandem repeats in intron 4	Reduced vaginal MBL levels	RVVC	Babula et al (2003 ), Giraldo et al (2007)
NLPR3	Length polymorphism	Impaired IL-1β production	RVVC	Lev-Sagie et al (2009)
PTPN22	R620W (rs2476601)	Unknown	Increased risk for CMC	Nahum et al (2008)
TLR1	R80T (rs5743611), S248N (rs4833095), I602S (rs5743618)	Decreased production of IL-1β, IL-6 and IL-8 after TLR1-TLR2 stimulation	Increased susceptibility to candidemia	Plantinga et al (2012)
TLR2	R753Q (rs5743708)	Decreased levels of IFN-γ and IL-8	Increased susceptibility to candidemia	Woehrle et al (2008)
TLR3	L412F (rs3775291)	Decreased IFN-γ levels	Increased risk for CMC	Nahum et al (2011, 2012)
TLR4	D299G (rs4986790), Y399I (rs4986791)	Increased IL-10 production	Increased susceptibility to candidemia	Van der Graaf et al (2006)

## Future developments

The current body of evidence has provided many new insights into the working mechanism of the anti-*Candida* immune response. These new insights can pinpoint novel potential targets for immunotherapy. For example, several studies have demonstrated a correlation between decreased IFN-γ levels and an increased risk for systemic *Candidiasis* (Johnson et al, 2012; Woehrle et al, 2008). A double-blind, randomized, placebo-controlled study is currently being performed using adjuvant IFN-γ therapy in sepsis. It would be also very relevant to try and reverse the immunoparalysis (Leentjens et al, 2012). This suggests that IFN-γ is a promising treatment option in sepsis-induced immune paralysis. We are currently investigating the efficacy of recombinant IFN-γ in patients with *Candida* sepsis.

Despite the significant progress of the last few years for uncovering susceptibility to fungal infections, there are still a significant number of *Candida* infections for which the environmental and/or genetic risk factors are not yet deciphered. Even more importantly, in spite of current treatment regimens, mortality rates associated with systemic infections are still very high, and in order to improve diagnostic- and treatment options, future efforts should be directed towards gaining more insight into the anti-*Candida* host immune response. This can be achieved in several ways. Discovering novel mutations that underlie monogenetic disorders associated with *Candida* infections can generate crucial information about a particular gene or protein, and the pathway in which this protein is involved. For example, the use of next generation sequencing and whole exome sequencing to discover *STAT1* mutations as a cause of CMC (van de Veerdonk et al, 2011), has also led in the understanding of its role for the generation of Th1 and Th17 responses and the anti-*Candida* host defense (Smeekens et al, 2011). This discovery can lead to novel approaches to the therapy of CMC, some of them being currently tested.

Of course, the list of existing monogenetic disorders is relatively small, as the majority of *Candida* cases are likely polygenic and/or multifactorial. In order to investigate this type of disorders other methods will have to be employed such as genome-wide association studies (GWAS), deep sequencing, and systems biology. We have recently used a combination of transcriptional analysis and functional genomics to demonstrate that type I IFNs play an important role in the anti-*Candida* host defense (Smeekens et al, 2013). Stimulation of circulating leukocytes with *C. albicans* led to a transcription profile with overrepresentation of genes from the type I IFN pathway. Subsequently, we showed that polymorphisms in these genes modify *Candida*-induced cytokine production and influence susceptibility to systemic *Candida* infections. Furthermore, validation studies showed that type I IFNs skew *Candida*-induced cytokine responses from Th17 toward Th1, while STAT1-deficient CMC patients display defective expression of genes in the type I IFN pathway. This ‘systems approach’, that integrates the information on anti-*Candida* host defense from several types of studies, provides information with respect to potential novel anti-*Candida* immune responses that may represent targets for immunotherapy. It is to be expected that an integration of efforts from immunology, genetics, microbiology and systems biology will represent the novel level of understanding of host defense against fungal (and other) pathogens, improving the outcome of these severe infections.

**Pending issues**

Integration efforts from immunology, genetics, microbiology and systems biology to increase the level of understanding of the host defense against fungal (and other) pathogens.

Design of novel immunotherapeutic strategies for an improved treatment.

Discovering novel mutations that underlie monogenetic disorders associated with *Candida* infections by GWAS or deep sequencing.
